# A case of swelling in the right maxilla

**DOI:** 10.4103/0973-029X.80010

**Published:** 2011

**Authors:** Revati S Deshmukh, Prasad Karande

**Affiliations:** *Department of Oral Pathology and Microbiology, Bharti Vidyapeeth University Dental College and Hospital, Dhankawadi, Katraj, Pune, India*

Rhinosporidiosis is an inflammatory disease endemic in India. It is characterized by hyperplastic polypoid lesions of the mucous membranes and is caused by Rhinosporidium seeberi. Identification of numerous globular cysts measuring upto 200 nm in diameter. Each of these cysts represents a thick walled sporangium containing numerous spores.

A 60-year-old female reported to Bharti Vidyapeeth Dental College and Hospital, Pune, with a chief complain of swelling in the right maxillary region since 2-3 months. She gave a history of fever few months back and had a habit of betel quid chewing since 40 years. She used to work in fields as a laborer.

## CLINICAL EXAMINATION

On clinical examination an intraoral swelling was noted, which was extending from first right maxillary premolar to first molar and was obliterating the buccal vestibule.

## RADIOLOGICAL EXAMINATION

On radiological examination unilocular radiolucency was noted, which was extending in the periapical region of first right premolar to periapical region of first molar. It was also involving the right maxillary sinus.

## HISTOPATHOLOGICAL EXAMINATION

### Under scanner view

Lesion lined by epithelium and underlying connective tissue was noted. It also showed some duct like spaces and some vacuolated areas in the underlying connective tissue.

### Under 10× magnification

**Figure 1:**
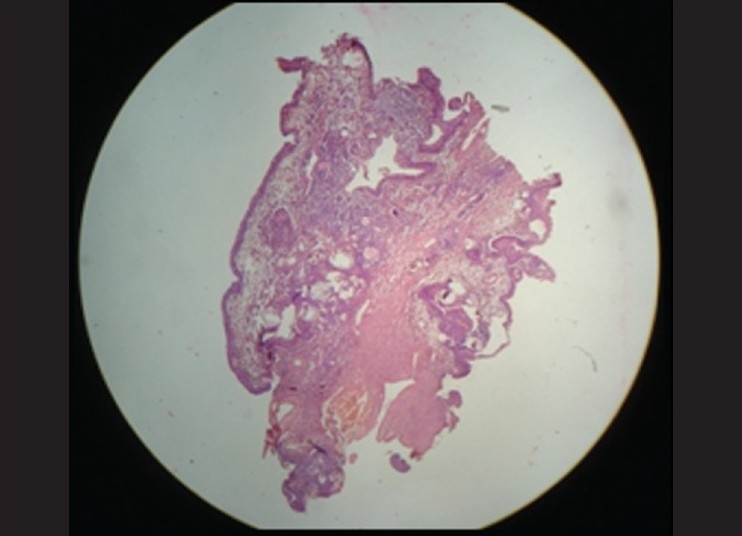
4× magnification showing cystic lining with inflammatory cells and globular cysts

**Figure 2:**
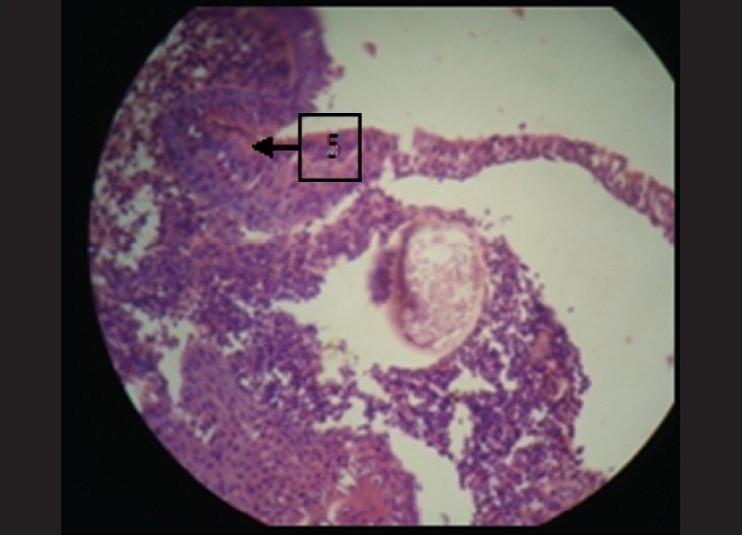
Squamous metaplasia under 10× magnification

**Figure 3:**
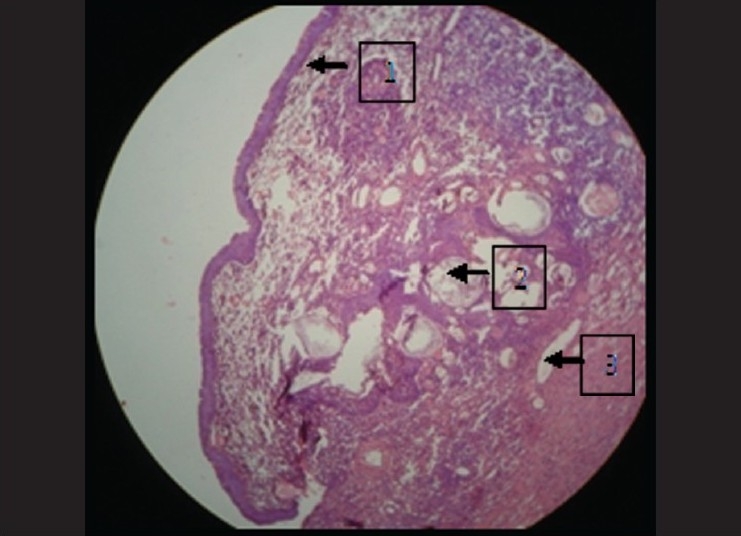
1) Pseudostratified columnar ciliated epithelium, 2) Squamous metaplasia, 3) Chronic inflammatory cells

**Figure 4:**
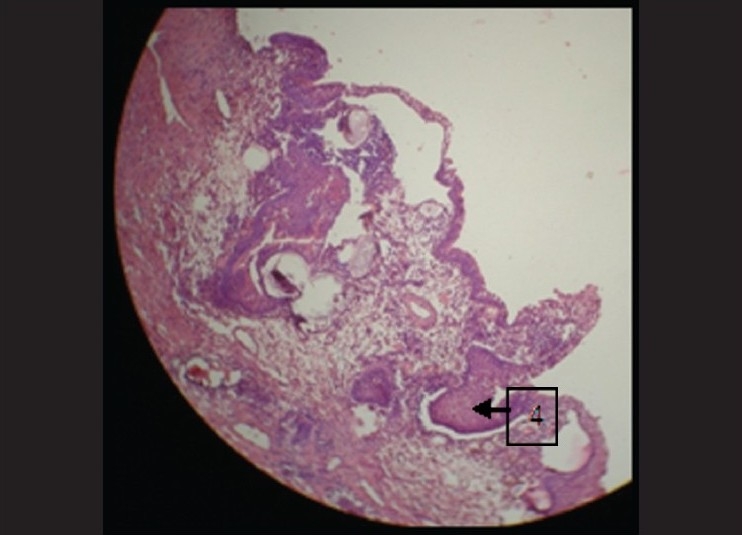
Large globular cysts surrounded by inflammatory cells under 4×

Epithelium was seen to be pseudostratified columnar ciliated in nature suggestive of maxillary sinus lining [Figure 1]. This epithelium at few places showed squamous metaplasia [Figure 2]. The underlying connective tissue showed dense chronic inflammatory infiltrate composed of lymphocytes, and plasma cells were evident which were concentrated around some vacuolated structures [Figure 3]. Few collagen fibers interspersed with fibroblasts and few blood vessels were seen which were hyalinized in nature [Figure 4]. Some areas also showed myxoid degeneration.

### Under higher magnification

**Figure 5:**
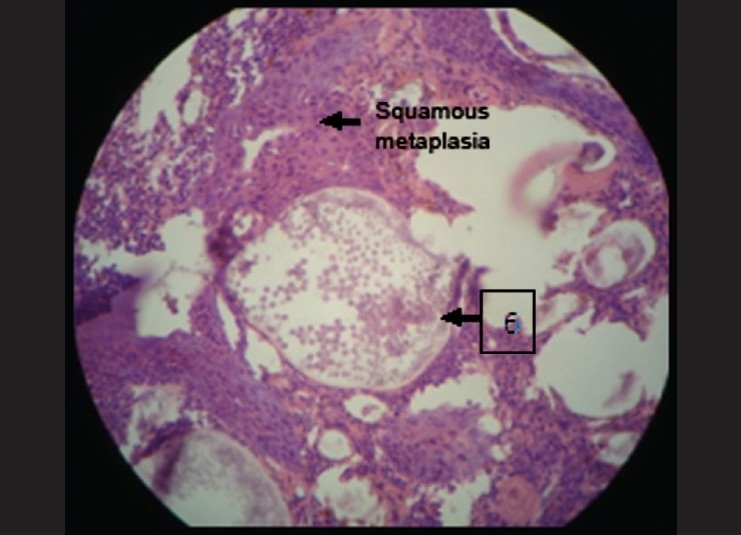
Sporangium with endospores

**Figure 6:**
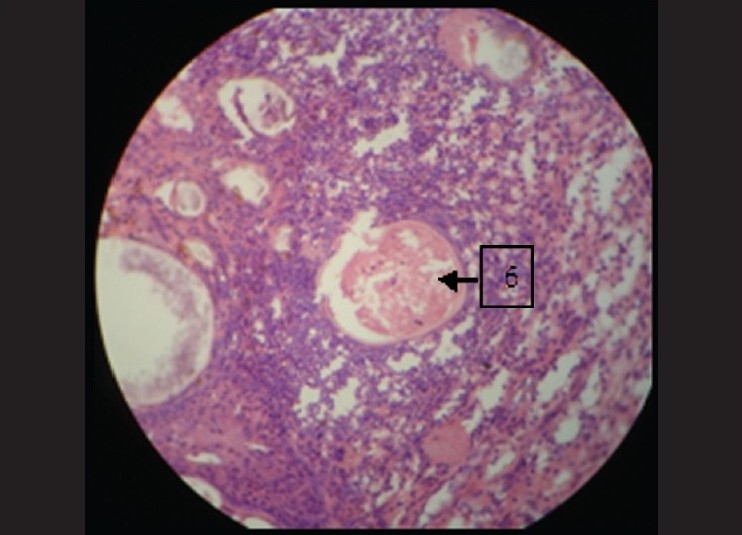
Globular cysts surrounded by heavy inflammatory cells

Epithelium clearly was seen to be pseudostratified columnar ciliated type. It also showed squamous metaplasia at many places [Figure 5]. In the connective tissue the vacuolated areas were seen to be having a hyalinized wall. Inside this wall there were few pale eosinophilic bodies. These structures were suggestive of sporangium filled with endospores [Figure 6]. These spores were intact at few places and seemed to burst open at other places. There was presence of chronic inflammatory cells chiefly composed of lymphocytes and plasma cells.

## DIAGNOSIS

Based on all the above histopathological features a final diagnosis of Rhinosporidiosis was given.

